# Supplementary material for: COZOID: contact zone identifier for visual analysis of protein-protein interactions
Source: BMC Bioinformatics. 2018 Apr 6;19:125. doi: 10.1186/s12859-018-2113-6 (PMC5889581; doi:10.1186/s12859-018-2113-6)
Supplement: Supplementary file 2 — Software build. Executable binary file of the software tool. (ZIP 118,784 kb) [file 12859_2018_2113_MOESM2_ESM.zip › cozoid/java/docs/beansbinding-1.2.1-doc.zip!/index-files/index-21.html]

U-Index (Beans Binding)


---


|  |  |  |
| --- | --- | --- |
| **Overview** \| Package \| Class \| Use \| **Tree** \| **Deprecated** \| **Index** \| **Help** | |  |
| **PREV LETTER**   **NEXT LETTER** | **FRAMES**    **NO FRAMES**     **All Classes** |  |


A B C D E F G H I J K L M N O P Q R S T U V W 

---


## **U**

**Unary()** - Method in class org.jdesktop.el.impl.parser.ELParser: **unbind()** - Method in class org.jdesktop.beansbinding.Binding: Unbinds this binding. **unbind()** - Method in class org.jdesktop.beansbinding.BindingGroup: Calls `unbind` on all bound bindings in the group. **unbindImpl()** - Method in class org.jdesktop.beansbinding.AutoBinding: **unbindImpl()** - Method in class org.jdesktop.beansbinding.Binding: Called by `Binding.unbind()` to allow subclasses to uninitiate binding. **unbindImpl()** - Method in class org.jdesktop.swingbinding.impl.AbstractColumnBinding: **unbindImpl()** - Method in class org.jdesktop.swingbinding.JComboBoxBinding: **unbindImpl()** - Method in class org.jdesktop.swingbinding.JListBinding: **unbindImpl()** - Method in class org.jdesktop.swingbinding.JTableBinding: **unbindUnmanaged()** - Method in class org.jdesktop.beansbinding.Binding: A protected version of `Binding.unbind()` that allows managed subclasses to unbind without throwing an exception for being managed. **UNREADABLE** - Static variable in class org.jdesktop.beansbinding.PropertyStateEvent: Used to indicate that a particular value is unreadable. **UNRESOLVABLE\_RESULT** - Static variable in class org.jdesktop.el.ELContext: **unsetSourceUnreadableValue()** - Method in class org.jdesktop.beansbinding.Binding: Unsets the value of the `sourceUnreadableValue` property by clearing the value and setting the value of the `sourceUnreadableValueSet` property to `false`. **UpdateLineColumn(char)** - Method in class org.jdesktop.el.impl.parser.SimpleCharStream

---


|  |  |  |
| --- | --- | --- |
| **Overview** \| Package \| Class \| Use \| **Tree** \| **Deprecated** \| **Index** \| **Help** | |  |
| **PREV LETTER**   **NEXT LETTER** | **FRAMES**    **NO FRAMES**     **All Classes** |  |


A B C D E F G H I J K L M N O P Q R S T U V W 

---
